# Helpful family climate moderates the relationship between perceived family support of ADHD symptoms and depression: a conditional process model

**DOI:** 10.1186/s40359-021-00615-5

**Published:** 2021-07-28

**Authors:** Pichaya Pojanapotha, Chiraphat Boonnag, Sirinut Siritikul, Sirikorn Chalanunt, Pimolpun Kuntawong, Nahathai Wongpakaran, Tinakon Wongpakaran

**Affiliations:** 1 Faculty of Medicine, Chiang Mai University, Chiang Mai, Thailand; 2 Department of Psychiatry, Faculty of Medicine, Chiang Mai University, 110 Intawaroros Rd., T. Sriphum, A. Muang, Chiang Mai, 50200 Thailand

**Keywords:** Adult ADHD, Undergraduate, Family support, Depression, Family climate

## Abstract

**Background:**

Symptoms of attention deficit hyperactivity disorder (ADHD) are commonly comorbid with depression This study aimed to examine the relationship between ADHD symptoms and depression through perceived family support and to explore whether the magnitude of the relationship depended on the type of family climate of medical students.

**Methods:**

This cross-sectional study was conducted among 124 first year medical students in Thailand. Participants completed questionnaires on ADHD symptoms, depression, perceived family support, and 9 types of family climate. The questionnaires included the Adult ADHD Self-Report Scale Screener, Patient Health Questionnaire-9, and revised Thai Multidimensional Scale of Perceived Social Support. Mediational analysis was adopted to examine the mediating role of perceived family support in the relationship between ADHD symptoms and depression, while moderation analysis was applied to examine the extent of the relationship depending on family climate.

**Results:**

The relationship between ADHD symptoms and depression was moderate. Perceived family support partially mediated this relationship after controlling for age and sex. Among the types of family climate, only helpful family climate was a significant moderator of perceived family support and depression. The moderated mediation model increased the variance in depression from 17% by the mediation model to 21%. However, follow-up conditional mediational analysis showed that the indirect effect of ADHD symptoms on depression via perceived family support was not significant and that this effect did not vary linearly as a function of helpful family climate.

**Conclusion:**

The findings of the study revealed that poor family support might be one risk of developing depression in the context of ADHD symptoms. Further study on providing intervention concerning family support among those with ADHD symptoms should be warranted. In addition, a study on helpful family climate in a larger sample size, in other populations, and in a longitudinal fashion for a more robust conclusion is encouraged.

## Background

Attention-deficit/hyperactivity disorder (ADHD) is a neurodevelopmental disorder that affects many areas of life from childhood to adulthood [1, 2]. ADHD symptoms include impairment in executive functions, such as self-regulation, time management, organization, working memory, planning, and cognitive flexibility, which are usually followed by academic failure, undermined work performance, and social problems [3–5]. On the whole, it leads to poor self-esteem and depression [6, 7].

ADHD is rarely acknowledged among adults, especially those who have never been treated as children [8]. A study has shown that 5.5% of adults reported received a diagnosed of ADHD, and most (72%) had the diagnosis after the age of 18 [9]. ADHD symptoms can affect learning and work efficiency and decrease the likelihood of achievement and happiness in many aspects of life [10–12], including quality of life [13, 14].

ADHD is commonly comorbid with depression. According to Pehlivanidis, psychiatric disorders, especially depressive disorders, are highly prevalent comorbidities of newly diagnosed adult ADHD [15]. The prevalence of depression in ADHD ranges from 18.6 to 53.3% [1, 16–18]. A longitudinal study showed that 17% of adults with a diagnosis of ADHD had depression as a comorbidity [19]. In addition to depression, among young adults, e.g., medical students, ADHD symptoms were also found to be significantly associated with alcohol consumption and suicidal behavior [20–22]. The mechanism of developing depression in ADHD is not fully understood; it may be attributed to various factors. Some studies have shown associations with self-esteem, perceived social support, and family climate [6, 23–26].

The treatment for ADHD includes both pharmacologic and psychosocial aspects. Family climate is one of the most important psychosocial factors associated with behavioral outcomes or symptoms. No systematic methods can describe and measure family climate. The ideal family climate is warm and stable, as this may help support children when they encounter challenges in life [27]. Some have described it using terms such as ‘democratic’, ‘authoritarian’, ‘extra-protection’ and ‘neglect’, while some use broader terms such as ‘favorable’ [28] or ‘aversive’ emotional climate [29]. To be able to classify family climate as positive or negative would be useful for clinical applications. Related work has shown that negative family climate is associated with externalizing behavior [30]. Both family issues and development of ADHD symptoms can have a bidirectional effect; for example, child impulsivity or activation can trigger parental hostility, which may lead to continuation of ADHD in the child [31–33]. A less favorable family climate is associated with ADHD symptoms [28]. Nevertheless, while most studies have analyzed the relationship between negative family environment and ADHD symptoms, positive environmental factors have often been ignored.

Perception of the family climate is our subject of interest because it affects an individual’s emotions, thinking, and behavior, regardless of what the real family climate is [30]. The ideal family climate should be warm and stable, as it may help support the child when they encounter challenges in life [27]. However, no systematic methods exist to describe and measure family climate. Studies investigating positive climate have illustrated it by family cohesion and communication [34] and warmth, supportiveness, and interpersonal engagement of the family [35], while those investigating negative family climate demonstrated it by undesirable family environments, emotionally unstable parents [36], authoritative parenting styles [37], highly emotional expressions and overinvolvement [30], and lack of emotional support, irritability, and intrusiveness [38]. Family climate is an essential predictor of youth depression [39], particularly negative family climate [40].

Based on the findings of these studies, ADHD symptoms, perceived family support, and family climate have an influence on depression among young adults. The effect is not only direct; an indirect effect of ADHD symptoms on depression via many factors has also been found. These mediators include the parent–child relationship, parenting stress, lack of social support, exposure to community violence, peer problems, and victimization [41–45]. However, indirect effects of ADHD symptoms on depression mediated by perceived family support and family climate have never been reported.

This study aimed to examine the relationship between ADHD symptoms and depression through perceived family support and to investigate whether the magnitude and direction of the relationship depended on family climate. Based on the findings of previous studies, we hypothesized that ADHD symptoms would positively relate to depression, while poor perception of family support would be more likely to be associated with increased depression. Positive family climate should be negatively associated with depression while negative family climate should be positively associated with depression.

## Methods

### Study population and procedure

This study surveyed 124 participants who were first year medical students at Chiang Mai University, Thailand. The sample was recruited in 2017 using convenience sampling. Each participant gave written informed consent before completing the questionnaires, which included sociodemographic data and information relating to the parental and family environment, the Adult ADHD Self-Report Scale (ASRS) screener, perceived family support questionnaire, and depressive symptom questionnaire. Ethics approval was obtained from the Faculty of Medicine, Chiang Mai University, Thailand, before taking any further steps in the research.

### Instruments

#### General demographic data

The self-reported questionnaires consisted of items on sociodemographic and health-related characteristics, including sex, age, underlying diseases, and family income. They also included nine factors that characterized family climate as perceived by the participant: quiet-lonesome, vivacious, chaotic, ignoring/detached, helpful, intrusive, warm-relaxing, distant, and uncomfortable/tense. In addition, information was collected on the parents’ education, occupation, and history of underlying physical and mental illness.

#### Adult ADHD Self-Report Scale (ASRS) screener V1.1

ASRS, developed by Kessler et al., is a six-item self-rated questionnaire measuring ADHD symptoms, and includes four items measuring inattention (items 1–4), and two items measuring hyperactivity (items 5–6) [46]. These six items are (1) trouble wrapping up the final details of a project, (2) difficulty getting things in order, (3) problems remembering appointments or obligations, (4) avoiding or delaying getting started, (5) fidgeting or squirming with hands or feet when sitting for long, and (6) feeling overly active and compelled to do things. The responses range from 0 to 4 (never to very often). Subjects reporting higher total score, present higher levels of ADHD symptoms. An individual with at least four symptoms is classified as having ADHD. The Thai version of the ASRS-v1.1 screener has been validated and used for screening adult ADHD; it shows a sensitivity of 0.93 and a specificity of 0.71 [47, 48]. Cronbach’s alpha for the study sample was 0.80.

#### Patient health questionnaire (PHQ)-9

The PHQ-9 is a nine-item self-reporting questionnaire measuring the extent to which an individual has experienced depressive symptoms over the past two weeks [49]. The four-response Likert scale ranges from 0 (not at all) to 3 (nearly every day). Subjects reporting higher total score, present higher levels of depressive symptoms. Cronbach’s alpha for the Thai version of the PHQ-9 is 0.79, and a positive association was noted between the PHQ-9 and the Hamilton Rating Scale for depression (*r* = 0.56, *p* < 0.001) [50]. Cronbach’s alpha for the study sample was 0.85.

#### Revised-Multidimensional Scale of Perceived Social Support (rMSPSS)

The rMSPSS tool measures the extent to which an individual has experienced support by significant others (SO), friends (FR), and family (FA) [51]. It comprises 12 questions, with responses rated on a seven-point Likert scale ranging from very strongly disagree (0) to very strongly agree (6). Subjects receiving higher scores, present higher levels of perceived social support. The revised Thai version demonstrated good psychometric properties [52]. In this study, only the family subscale was used; its Cronbach’s alpha was 0.88.

### Statistical analysis

Descriptive statistics—frequency, percentage, and mean and standard deviation—were obtained for sociodemographic, ADHD-symptom and mental health-related data. Mean and standard deviation were calculated for continuous data. In the case of discrete data, such as sex, the amount and percentage were obtained. Between group differences and correlations were analyzed using independent *t*-tests and Pearson’s correlation.

**Fig. 1:**
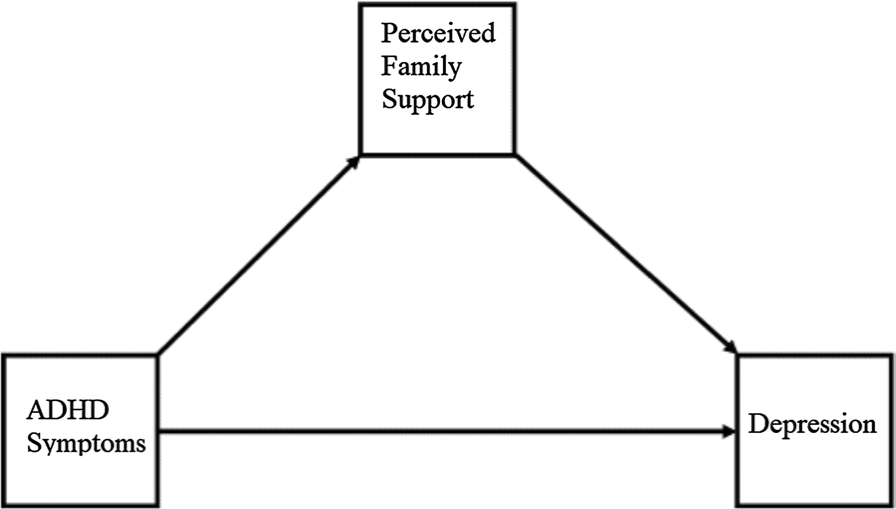
Mediation model

**Fig. 2:**
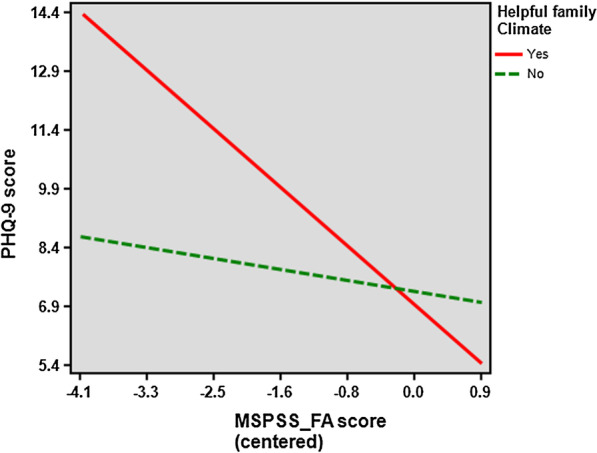
Relationship between PHQ-9 and MSPSS (family) scores for family climate perceived as helpful (yes) or as not helpful (no). *MSPSS_FA* Multidimensional Scale of Perceived Social support (family subscale), *PHQ-9* nine-Item Patient Health Questionnaire

In the analysis of the mediation and moderation models, we began by examining the magnitude of the relationships between ADHD symptoms, depression, perceived family support, and family climate using zero-order correlations. For mediation analysis, we used the methods discussed by Hayes [53] to examine the relationship between ADHD symptoms and depression through perceived family support (Fig. 1). For moderation analysis, we conducted tests to determine which type of family climate was a significant moderator of the relationship between ADHD symptoms and perceived family support, ADHD symptoms and depression, and perceived family support and depression. We found that only the family climate perceived as helpful was a moderator of the relationship between perceived family support and depression (Fig. 2). Therefore, we used Hayes’s Model 14, a moderated mediation analysis that simultaneously tests how a relationship between an antecedent variable (X) and an outcome variable (Y) depends on the level of the moderator. In this case, we tested a moderated mediation model in which depression was regressed on ADHD symptoms through perceived family support. Model 14 further specifies that the effect of the hypothesized mediating or antecedent variable (perceived family support) on the outcome or consequent variable (depression) is conditional on the moderating variable (helpful family climate). Significant interaction (perceived family support × helpful family climate) was examined by visualizing predicted values of depression-centered scores with the presence or absence of helpful family climate [53]. We used resampling or bootstrapping and the product of coefficients as suggested by Preacher and Hayes when conducting mediation and moderation analyses [53, 54]

We used PROCESS v3.4, an add-on statistical analysis for SPSS created by Hayes [55], for all mediation and moderation analyses. For interpretation, PROCESS provides standard errors, *p*-values, confidence intervals for the direct effect coefficients, and bootstrap confidence intervals for conditional indirect effects and for conditional indirect effects pairwise contrasts. Confidence intervals that do not straddle zero are indicative of statistical significance. For all the analyses, the level of significance was set at *p* < 0.05. All statistical analyses were performed using the program IBM SPSS 22.0.

## Results

As shown in Table 1, 62.9% of the participants were female, with an age range of 17 to 21 years. None reported any mental illness or being under any medication. Both parents had an average of 15 years of education. Slightly over one half of the participants were firstborn. The median number of siblings was 2 (range = 1 to 6). Most participants described the family they grew up in as enjoyable (83.9%), helpful (85.5%), and warm (83.9%).

**Table 1:** Sociodemographic characteristics of the participants

Variable	n (%) or mean ± SD
Male	46 (37.1)
Age (years)	18.78 ± 0.74
Father’s years of education	14.98 ± 5.6
Father’s mental illness	1 (0.8)
Mother’s years of education	14.71 ± 5.7
Mother’s mental illness	0
Single child	18 (14.5)
Firstborn	65 (52.4)
Last born	46 (37.4)
Number of siblings	2.1 ± 0.79
*Family climate*	
Quiet-lonesome	5 (4)
Enjoyable	104 (83.9)
Chaotic	6 (4.8)
Ignoring/detached	1 (0.8)
Helpful	106 (85.5)
Intrusive	8 (6.5)
Distant	0 (0)
Warm-relaxing	104 (83.9)
Uncomfortable/tense	8 (6.5)

ADHD symptoms, PHQ-9 scores, and MSPSS_FA scores were significantly related to each other, while no significant correlations between family climate and ADHD symptoms were found (Table 2). As expected, positive climate factors—enjoyable, helpful, and warm—positively correlated with MSPSS_FA scores, but negatively correlated with the PHQ-9 scores, while negative family climate factors positively correlated with the PHQ-9, but negatively correlated with the MSPSS_FA.

**Table 2:** Correlation matrix between family climate and clinical variables

Variables	M (SD)	1	2	3	4	5	6	7	8	9	10	11
Quiet-lonesome	0 (0)	1										
Enjoyable	1 (0)	− .467^**^	1									
Chaotic	0 (0)	− .046	− .514^**^	1								
Ignoring/detached	0 (0)	− .018	− .206^*^	.400^**^	1							
Helpful	1 (0)	.084	.566^**^	− .227^*^	− .219^*^	1						
Intrusive	0 (0)	− .054	− .063	.247^**^	− .024	− .637^**^	1					
Warm-relaxing	1 (0)	− .022	.523^**^	− .105	− .206^*^	.628^**^	− .153	1				
Uncomfortable/tense	0 (0)	.113	− .153	.247^**^	.343^**^	− .265^**^	.332^**^	− .599^**^	1			
ADHD symptoms	10.1 (3.5)	− .122	− .072	.137	.013	− .164	.145	.029	.016	1		
MSPSS_FA score	6.1 (1.0)	− .096	.320^**^	− .287^**^	− .029	.270^**^	− .224^*^	.284^**^	− .299^**^	− .264^**^	1	
PHQ-9 score	7.2 (3.9)	.128	− .309^**^	.086	.089	− .218^*^	− .005	− .235^**^	.106	.268^**^	− .368^**^	1

M(SD) = mean (standard deviation); from Quiet-lonesome to Uncomfortable/tense variables, the values are median (interquartile range)

*p < 0.05; **p < 0.01

*MSPSS_FA* Multidimensional Scale of Perceived Social support (family subscale), *PHQ-9* Nine-Item Patient Health Questionnaire, Distant family climate was omitted from analysis

### Mediation model

The mediation model showed that both ADHD symptoms and perceived family support had a significant effect on depression. Perceived family support reduced the effect of ADHD symptoms from 0.07 (*t* = 3.022, *p* = 0.003) to 0.058 (*t* = 2.141, *p* = 0.0343). ADHD symptoms appeared to have an indirect, rather than a direct effect, on depression through perceived family support.

**Fig. 3:**
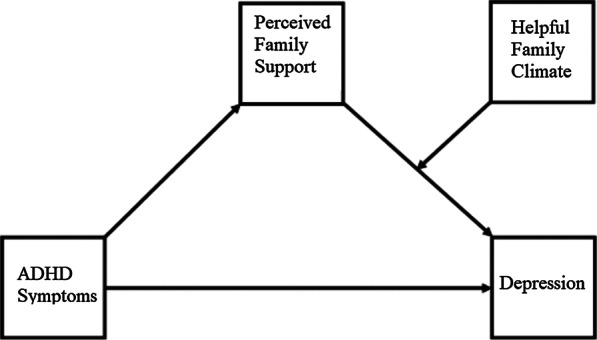
Moderated mediation model

Figure 2 shows the relationship between PHQ-9 and MSPSS_FA scores based on the presence or absence of a family climate perceived as helpful. Moderational analysis showed that the slope for the presence of a helpful family climate was -1.817 (95% CI = -2.579, -1.054; standard error of slope = 0.38; degrees of freedom = 111; *t* = -4.721, *p* < 0.0001); while the absence of a helpful family climate was -0.336 (95% CI = -2.151, 1.479; standard error of the slope = 0.92; degrees of freedom = 111; *t* = -0.366, *p* = 0.357). Therefore, for participants perceiving their family climate as being helpful, the correlation between perceived family support and depression was significantly negative (Fig. 3).

Moderation analysis showed that perceived family support yielded significantly different effects on depression for the group that perceived a helpful family climate (effect = 0.0349, BootSE = 0.0191, BootLLCI = 0.0036, and BootULCI = 0.0786).

The MSPSS_FA significantly mediated the relationship between ADHD symptoms and the PHQ-9 (*t* = -0.3.716, *p* = 0.0003; Table 3), while a significant moderation effect of helpful family climate on decreased depression was also observed (*t* = -2.129, *p* = 0.035). Compared with the mediation model, the moderated mediation model increased *R*^2^ from 16.7 to 20.8%. A conditional process model produced the effect of 0.033 for the index of moderated mediation, with a 95% bootstrap confidence interval between -0.0053 and 0.0944. As this confidence interval straddles zero, this provided evidence that the indirect effect of ADHD symptoms concerning depression through perceived family support was not moderated by helpful family climate. In other words, as the confidence interval straddled zero, we could not claim definitively that the indirect effect varied linearly as a function of helpful family climate; hence, no moderation of mediation was indicated.

**Table 3:** The results of the mediation model and moderated mediation model

PHQ-9	Coefficient	SE	*t*	*p *value	LLCI	ULCI
*Mediation model*						
ADHD symptoms	0.058	0.027	2.141	0.0343	0.004	0.112
MSPSS_FA	− 1.241	0.334	− 3.716	0.0003	− 1.901	− 0.58
*R*^*2*^	0.1671					
Indirect effect	0.084	0.043			0.012	0.177
*Moderated mediation model*						
ADHD symptom	0.047	0.027	1.727	0.0867	− 0.007	0.100
MSPSS_FA (M)	− 0.096	0.594	− 0.161	0.8726	− 1.272	1.081
Helpful family climate (W)	7.432	4.160	1.787	0.0766	− 0.806	15.669
M* W	− 1.5331	0.7201	− 2.129	0.0353	− 2.959	− 0.107
*R*^*2*^	0.2084					
Index of moderated mediation	0.033	0.026			− 0.005	0.094

*SE* standard error, *LLCI* lower level of confidence interval, *ULCI* upper level of confidence interval

## Discussion

This study aimed to examine the extent to which perceived family support and family climate contributed to decreased depression among medical students. Despite the increase (from 17 to 21%) in the variance in depression explained by adding the variables perceived family support and helpful family climate, the indirect effect of ADHD symptoms through perceived family support was not significantly linearly contingent on helpful family climate. In the mediation model, depression was explicitly influenced by the direct effect of perceived family support and the mediating effect of ADHD symptoms. The impact of helpful family climate was not large, despite having a moderating effect on perceived support for depression. The main reason for the nonsignificance may be attributed to the fact that the sample size was not sufficiently large to capture small differences. Another possible reason may be that the family climate data collected focused on the respondent’s experience rather than the current family atmosphere, which should have a greater interaction effect with the current feeling of family support. This finding was in line with that of a related study [30].

Perceived family support intuitively positively correlates with positive family climate, i.e., enjoyable, helpful, and warm, but negatively correlates with negative family climate, i.e., chaotic and intrusive. However, our analysis showed that only helpful family climate was a significant moderator in the moderation model. These findings are supported by Wüstner et al. in that no associations were observed initially between family climate and ADHD but at a later time [45]. However, the main emphasis of the two studies differed; this study focused on depression, while Wüstner et al. focused on ADHD symptoms. Moreover, the current study found an association between perceived family support and ADHD symptoms and depression, whereas Wüstner et al. found that social support reduced the association between strong parental mental health problems and strong ADHD symptoms. Further study is worth pursuing to test the longitudinal effect of perceived family (or social) support on the relationship between ADHD symptoms and depression.

Notably, regarding family climate, related studies have suggested that negative family relationships could increase the incidence of ADHD symptoms [56], and that ADHD symptoms had an impact on family climate. This constitutes a vicious cycle [33]. Oddly enough, the present study did not find a relationship between either positive or negative family climate and ADHD symptoms; this could possibly have been because the questionnaire was insufficient to capture the significant relationships found in related studies [30].

### Strengths and limitations

To the best of our knowledge, this study is one of the first concerning the importance of perceived family support and helpful family climate on depression in adult ADHD. Further investigations based on these results are recommended. Given that ADHD is highly prevalent and causes significant impairments in almost all areas of life, our findings have important implications for prevention and clinical practice. In addition to family-based interventions, future prevention and early intervention programs should focus on the availability of good social support and enhancing social skills, particularly among children of mentally ill parents, to reduce risks and prevent the onset of ADHD symptoms.

This study encountered several limitations. First, the sample size was small and the participants were all first year medical students, which cannot represent the general medical student body. We used self-reported data to assess ADHD symptoms without interview confirmation, yielding a disproportionately high prevalence. We did not evaluate conditions highly comorbid with ADHD, including bipolar disorder, anxiety disorder, and personality disorder. Future studies are required to further understand ADHD symptoms and mental health-related conditions among medicals students. In addition, this study lacked information about parental ADHD, which may be related to negative family climate.

## Conclusion

The findings of the study revealed that poor family support might be one risk of developing depression in the context of ADHD symptoms. Further study on providing intervention concerning family support among those with ADHD symptoms should be warranted. In addition, a study on helpful family climate using a larger sample size, as well as in other populations, and in a longitudinal fashion for a more robust conclusion is encouraged.

## Data Availability

The datasets used and/or analyzed during the current study are available from the corresponding author on reasonable request.

## Abbreviations

ADHD: Attention deficit hyperactivity disorder
ASRS: Adult ADHD Self-Report Scale
MSPSS-FA: Multidimensional Scale of Perceived Social Support—Family
PHQ-9: 9-Item Patient Health Questionnaire
